# Editorial: Hallmark of cancer: Reprogramming of cellular metabolism

**DOI:** 10.3389/fonc.2022.1126913

**Published:** 2023-01-11

**Authors:** Baljinder Kaur, Yahya Sohrabi, Abhinav Achreja, Michael P. Lisanti, Ubaldo Emilio Martinez-Outschoorn

**Affiliations:** ^1^ Systems Biology Laboratory, Department of Biotechnology & Food Technology, Punjabi University, Patiala, India; ^2^ Department of Cardiology I-Coronary and Peripheral Vascular Disease, Heart Failure, University Hospital Münster, Westfälische Wilhelms-Universität, Münster, Germany; ^3^ Department of Medical Genetics, Third Faculty of Medicine, Charles University, Prague, Czechia; ^4^ University of Michigan, Ann Arbor, MI, United States; ^5^ Translational Medicine, School of Science, Engineering and Environment, Biomedical Research Centre, University of Salford, Greater Manchester, United Kingdom; ^6^ Thomas Jefferson University, Philadelphia, PA, United States

**Keywords:** cancer, homeostasis, diagnosis, biomarker, dysbiosis

Growing evidence suggests that cell metabolism alterations represent a critical hallmark for majority of human cancer and tumors. One of the major differences between normal cells and cancer cells is relate to their growth and proliferation rate that require elevation in cell metabolism. Cancer cells undergo several important specific metabolic reprogramming such as increased aerobic glycolysis, pH deregulation, enhanced level of reactive oxygen species (ROS) and lipid metabolism dysregulation (Neagu et al.; Stine et al.). Studying all of these cellular deregulations may lead to identification of therapeutic targets. This special issue, presenting a compendium of eight articles, displays the role of metabolic reprogramming in development and progression of cancers, along with the underlying importance of metabolic compartmentalization within the cancer tissue. Furthermore, notable findings on early stage cancer diagnosis and novel drug targets in the broad domain of cancer metabolism are also presented as described below.

## Reprogramming of cellular metabolism

Fatty acid homeostasis plays pivotal role in the development and progression of cancers. The expression of enzymes involved in fatty acid and sterol metabolism is predominantly regulated by sterol regulatory-element binding proteins (SREBPs). Among three SREBP proteins, SREBP1a is the strongest transcription factor and its expression is restricted to rapidly proliferating cells, including cancer cells. The SREBPs are rapidly degraded by Fbw7 ubiquitin ligase dependent ubiquitin-proteasome system. Therefore, Fbw7 loss of function mutations stabilizes SREBP1a in cancer cells, which further augments the expression of SREBP target genes to promote lipid biosynthesis for the rapid proliferation of cancer cells. Interestingly, the inactivation of SREBP1 in cancer cells attenuates their proliferation through the inhibition of AKT pathway and the depletion of cholesterol (Bengoechea-Alonso et al.).

The reprogramming of fatty acid metabolism is also observed in the case of glioblastoma cancer, wherein it is mainly correlated with the enhanced expression of stearoyl CoA desaturase and/or fatty acid desaturase 2. Disruption of their activities breaks down fatty acid homeostasis in the cancer cells thus allowing palmitate accumulation that synergizes with anti-cancer agent temozolomide. This disruption was shown to augment temozolomide efficacy and induce death in glioblastoma cells by favouring the accumulation of saturated fatty acids such as palmitate (Parik et al.).

The alteration of the anaplerosis pathway mediated by pyruvate carboxylase shifts reliance of the cancer cells to other pathways that can be targeted to inhibit their proliferation and migration, accompanied by apoptotic induction. To prove this, CRISPRCas9 technique was used to generate pyruvate carboxylase knockout (PC KO) colon cancer cell line. The overexpression of PC was associated with staging, metastasis and poor survival of colorectal cancer patients. The PC KO HT-29 cells displayed growth deficient phenotype with apoptotic induction and inhibition of key lipogenic enzymes such as acetyl-CoA carboxylase-1 and fatty acid synthase. Furthermore, PC KO HT-29 cells become more sensitive to cytotoxic agents such as 5-fluorouracil and glutaminase inhibitor, CB-839 (Ngamkham et al.).

## Metabolic compartmentalization and crosstalk

The metabolic reprogramming was also reported in the case of ovarian cancer patients. It was demonstrated that the energy requirements of cancer cells are met by elevated glycolysis (lactic acid) and TCA cycle intermediates (malic acid, fumaric acid). These are accompanied by accumulation of glutathione and polyunsaturated fatty acids (linoleic acid) and depletion of saturated fatty acids (palmitic acid) that help overcome oxidative stress within the cancer tissue. The levels of alanine, aspartic acid, cysteine, glutamic acid, leucine, and proline decrease in plasma and urine samples as compared to the malignant ovarian tissue. Additionally, plasma displays elevated levels of fatty acids such as stearic acid, EPA, and arachidonic acid, while TCA cycle intermediates (succinic acid, citric acid, and malic acid) are more concentrated in the urine (Zhong et al.).

A study conducted on aerodigestive tract (ADT) cancers revealed the occurrence of metabolic compartmentalization within the cancerous tissue. An upregulation of the markers of oxidative phosphorylation such as monocarboxylate transporter 1 and translocase of the outer mitochondrial membrane 20 was observed in carcinoma cells. On the other hand, cancer-associated fibroblasts (CAFs) display upregulation of monocarboxylate transporter 4 (MCT4) and downregulation of isocitrate dehydrogenase 3a. The genetic depletion of CAF associated MCT4 decreases proliferation and survival of carcinoma cells by nutrient and energy depletion. Therefore, it can be concluded that the upregulation of MCT4 in CAFs drives aggressiveness in ADT cancers (Domingpo-Vidal et al.).

## Cancer diagnostics

A non-invasive method of diagnosing lung cancer at an early stage was proposed. In this pilot study, a diagnostic procedure based on the detection of important cancer biomarkers in sputum and breath condensate of early-stage non-small cell lung cancer patients before and after surgical resection (SR) was proposed. Enhanced levels of glucose, adenosine monophosphate, and N1, N12- diacetylspermine were reported in the sputum samples post-SR. These biomarkers could also be used for the further exploration of novel treatment regimens with better efficiencies (Ahmed et al.).

## Novel drug targets

The proliferating cancer cells are smart enough to meet their nutritional and energy requirements through diverse mechanisms such as metabolic plasticity or epigenetic remodelling. Therefore, the metabolic-epigenetic axis has recently become an interesting proposition to offer valid solutions for prognosis and treatment of cancers. The epigenetic remodelling of cancer cells is considered to be induced by nicotinamide N-methyltransferase (NNMT) enzyme that controls DNA methylation patterns and nucleosome phasing. Experimental observations have confirmed the overexpression of NNMT in cancerous tissues as well as body fluids of the cancer patients including serum, urine, and saliva. As NNMT knockdown could reverse cancer progression by controlling metabolic-epigenetic remodelling, therefore, NNMT is proposed as a potential candidate for developing diagnostic reagents and effective chemotherapeutic agents (Li et al.).

NADH:ubiquinone oxidoreductase subunit C1(NDUFC1) is a potential target for proposing novel anti-cancer therapy against hepatocellular carcinoma (HCC). It was reported for the first time that NDUFC1 is an independent risk factor of HCC. Enhanced expression of NDUFC1 in HCC cells is positively correlated with poor prognosis of the disease and reduced overall survival. It promotes tumor progression through inhibition of mitochondrial complex-I and up-regulation of ROS via. modulation of cancer related pathways viz. p53/PI3K/Akt/mTOR. An *in vitro* study, conducted on HCC cell lines with NDUFC1 gene knockout, has revealed that the downregulation of NDUFC1 suppressed migration and invasion of HCC cells (Han et al.).

Altogether, metabolic pathways, regulatory genes and enzymes are the therapeutic targets to disable cancer cells proliferation and tumor progression. Current issue describes several pathways and molecules that may provide a therapeutic advantage.

## Author contributions

BK prepared original draft of the editorial. AA- edited the original draft. YS edited original draft. ML edited original draft. UM-O guest editor of this Research Topic. All authors contributed to the article and approved the submitted version.
